# Difficult Respiratory Weaning after Cardiac Surgery: A Narrative Review

**DOI:** 10.3390/jcm12020497

**Published:** 2023-01-07

**Authors:** Davide Nicolotti, Silvia Grossi, Francesco Nicolini, Alan Gallingani, Sandra Rossi

**Affiliations:** 1 Department of Anesthesia and Intensive Care Medicine, Azienda Ospedaliero-Universitaria di Parma, Via Gramsci 14, 43126 Parma, Italy; 2 Department of Cardiac Surgery, Azienda Ospedaliero-Universitaria di Parma, Via Gramsci 14, 43126 Parma, Italy

**Keywords:** cardiac surgery, respiratory weaning, ventilation, tracheostomy

## Abstract

Respiratory weaning after cardiac surgery can be difficult or prolonged in up to 22.7% of patients. The inability to wean from a ventilator within the first 48 h after surgery is related to increased short- and long-term morbidity and mortality. Risk factors are mainly non-modifiable and include preoperative renal failure, New York Heart Association, and Canadian Cardiac Society classes as well as surgery and cardio-pulmonary bypass time. The positive effects of pressure ventilation on the cardiovascular system progressively fade during the progression of weaning, possibly leading to pulmonary oedema and failure of spontaneous breathing trials. To prevent this scenario, some parameters such as pulmonary artery occlusion pressure, echography-assessed diastolic function, brain-derived natriuretic peptide, and extravascular lung water can be monitored during weaning to early detect hemodynamic decompensation. Tracheostomy is considered for patients with difficult and prolonged weaning. In such cases, optimal patient selection, timing, and technique may be important to try to reduce morbidity and mortality in this high-risk population.

## 1. Incidence, Definition, and Risk Factors

Following cardiac surgery, most patients undergo rapid recovery of spontaneous breathing and extubation. However, between 2.6 and 22.7% of patients fail to wean from mechanical ventilation due to either cardiac or noncardiac problems [1,2]. The need for mechanical ventilation beyond 3 days has been related to a dramatic worsening of the outcome with a reported in-hospital mortality as high as 25–50% [3,4]. Long-term mortality and morbidity are also significantly increased in this subgroup [5]. Furthermore, prolonged ventilation leads to a significant increase in healthcare costs, up to 18 times compared to patients with early extubation [1,6].

Prolonged ventilation after surgery is not uniquely defined, and several studies use different definitions. However, the majority of investigators identify prolonged ventilation as the need for invasive ventilation (i.e., ventilation through an endotracheal tube or tracheostomy) for more than 48–72 h. A consensus defined three possible scenarios: easy weaning, difficult weaning, and prolonged weaning [7]. Easy weaning applies to most patients, where a single, successful attempt of separation from mechanical ventilation is performed; difficult weaning is defined when the first separation attempt fails, and more spontaneous breathing trials (SBTs) are needed before achieving extubation; prolonged weaning is when patients need more than three SBTs or more than 7 days of ventilation after the first SBT. Spontaneous breathing trials are performed by leaving the patient to breathe with little or no positive end-expiratory pressure. This can be done by using either pressure-support ventilation or a T-piece [8]. When a patient is unable to tolerate a trial of spontaneous breathing, this is aborted, and assisted mechanical ventilation is resumed. This is considered a “failed SBT”. When re-intubation is needed within 48 h after extubation, this is referred to as “failed extubation”.

Risk factors for prolonged mechanical ventilation following cardiac surgery have been identified and reviewed in previous publications, but only a few of them have been consistently reported. Factors of special interest to the cardiac surgery population include renal failure, type and timing of surgery, New York Heart Association (NYHA) and Canadian Cardiac Society (CCS) classes, the need for inotropic support, cardiogenic shock, or low cardiac output [9,10,11,12]. Other predictors of prolonged ventilation were identified in some studies but were not confirmed in other series, including blood products transfusion, body mass index (BMI), surgery and cardio-pulmonary bypass duration, and presence of atrial arrhythmias and chronic obstructive pulmonary disease [4,6,13,14,15].

Systolic dysfunction has long been considered a risk factor for difficult weaning after cardiac surgery; however, data are inconsistent. Indeed, Nozawa and colleagues found that patients with a left ventricle ejection fraction (LVEF) of less than 40% were 10 times more likely to require prolonged ventilation [15]. Moreover, Reddy et al. included LVEF of less than 30% in their logistic model to predict prolonged mechanical ventilation after cardiac surgery [16]. However, these findings were not confirmed by other studies [4,12]. Recent research is focusing more on the importance of diastolic, rather than systolic, failure in the pathogenesis of weaning-induced pulmonary oedema (WiPO, cf. Section 3.2) [17,18].

The aforementioned risk factors were used to identify patients at greater risk for difficult weaning. Many of these factors were also combined and different mathematical models were elaborated to predict the need for prolonged mechanical ventilation or tracheostomy after cardiac surgery but, to the best of our knowledge, none of them has been validated in prospective studies [1,13,15,19]. Table 1 resumes the most recent studies on risk factors for prolonged ventilation and difficult weaning after cardiac surgery.

## 2. Hemodynamic Effects of Weaning

**Figure 1:**
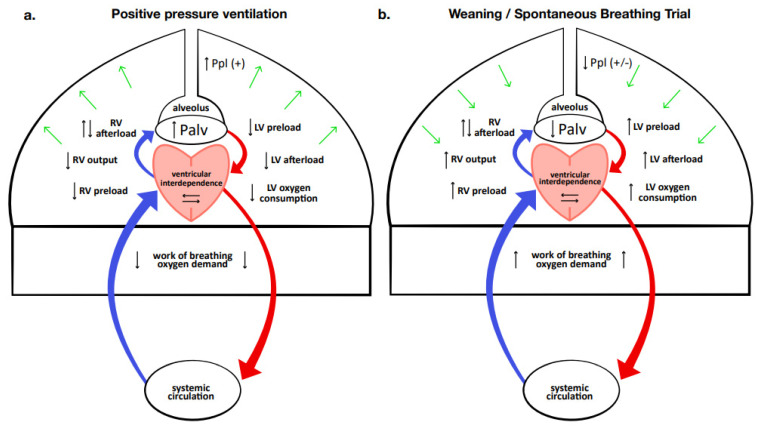
Heart–lung interaction, and hemodynamic effects of mechanical ventilation (**a**) and weaning/spontaneous breathing trial (**b**). During mechanical ventilation, the global effect of positive intrathoracic pressure is to reduce cardiac and global oxygen demand. During weaning and especially during spontaneous breathing trials, the reduction or abolition of positive intrathoracic pressure produces an increase in cardiac workload, while increased work of breathing, stress, and anxiety raise the global oxygen demand. Ppl, intrapleural pressure; Palv, alveolar pressure; RV, right ventricle; LV left ventricle.

The effects of positive pressure ventilation on the cardiovascular system have been well known for many years. There are numerous ventilation methods and weaning modes, but irrespective of the ventilator setting used, effects on cardiovascular function are dependent upon the changes generated in intrathoracic pressure and lung volume [20]. Indeed, the positive pressure generated inside the thoracic cavity produces different hemodynamic effects (Figure 1).

In the right heart, positive pressure causes a reduction in venous return and a variable effect on pulmonary resistance. In fact, the increase in lung volume and the compression of vascular structures generated by positive pressure usually leads to an increase in pulmonary vascular resistances; however, if positive pressure produces recruitment of previously non-ventilated areas, it may reduce hypoxic vasoconstriction, ultimately resulting in an overall reduction of vascular resistances. The net effect is usually a slight reduction in both the right ventricular workload and output.

The reduction in right ventricular output diminishes left ventricular filling, while the increased intrathoracic pressure reduces left ventricle end-diastolic volume, transmural pressure, and oxygen consumption. Furthermore, the support pressure delivered by the ventilator reduces the work of breathing, hereby decreasing global oxygen demand. Finally, the sedation that is usually associated lowers the sympathetic tone and consequently the peripheral vascular resistance and heart rate.

Overall, the combination of these hemodynamic alterations creates a perfect milieu that allows a failing heart to maintain balance, and it is therefore not surprising that positive pressure ventilation proves exceptionally effective in patients with acute or decompensated heart failure. Unfortunately, during the weaning from the ventilator, there is a progressive reduction of these effects, leading to an increase in heart preload and afterload, oxygen consumption, and sympathetic tone, due to a decrease in sedatives and emotional stress. For this reason, the weaning process can cause the development of acute ventricular dilation, myocardial ischemia, functional mitral regurgitation, and heart failure [3,10,17,21]. The decompensation of cardiac function may eventually manifest as WiPO, possibly leading to weaning failure [9,22].

## 3. Monitoring the Weaning and Assessing Causes of SBT Failure

As mentioned previously, weaning failure after cardiac surgery can be due to both cardiac and noncardiac causes. Therefore, a thorough evaluation of the underlying cardiac condition, as well as other factors, is mandatory. Some parameters were shown to be useful during the weaning process to assess the dynamic reaction of the cardiocirculatory system to the progressive reduction of ventilatory support. These indicators may therefore be helpful in anticipating the patient’s chances of successful weaning and guiding the weaning process. Useful parameters include pulmonary artery occlusion pressure (PAOP), extravascular lung water (ELW), echographic assessment of diastolic function, and biomarkers such as brain-derived natriuretic peptide (BNP) [9,18,23,24,25]. The predictive capability of these parameters and corresponding cut-off values vary among studies.

### 3.1. Pulmonary Artery Occlusion Pressure

We previously described the physiologic changes in the cardiocirculatory system during weaning (cf. Section 2). In the case of a failing heart, the inability of the cardiovascular system to cope with the growing workload results in an increase in the left ventricle filling pressure that can be estimated with a pulmonary artery catheter (PAC). In particular, the increase in pulmonary artery occlusion pressure (PAOP) can develop within a few minutes after disconnection from the ventilator [9]. A significant increase was also demonstrated during SBTs [26]. In their study in non-cardiac ICU, Cabello et al. found that patients tended to have a higher increment in PAOP during T-piece and PSV-ZEEP trials, compared to PSV-PEEP SBTs.

A cut-off value of 18 mmHg is classically used to identify pulmonary oedema of cardiac origin [9]. In small sample studies, patients with PAOP > 18 mmHg during SBT were significantly more likely to fail to wean, with a fourfold increased risk [27]. Nassar et al. also found that while PAOP > 18 mmHg strictly correlated with weaning failure, a lower cut-off of 15 mmHg did not show a significant correlation [28]. These data suggest a PAOP greater than 18 mmHg during SBT should be considered a warning sign that the patient is likely to fail weaning. However, the use of a single PAOP cut-off value in patients after cardiac surgery has some limitations and may be prone to confounding factors. Indeed, volume overload and positive intra-thoracic pressure can overestimate PAOP, as well as active expiratory effort from the patient, which is frequent during SBTs [26]. In this scenario, the measurement of the trans-pulmonary PAOP should be considered [29].

For patients that are not monitored with a PAC, cardiac ultrasound can provide a good, noninvasive alternative. Indeed, ultrasound measures—mainly E/E’—demonstrated a good capacity to detect a PAOP > 18 mmHg and to predict weaning failure (cf. Section 3.2), thus providing the clinician with a reliable, noninvasive tool to evaluate cardiac dysfunction during SBT [28,30,31].

In summary, PAOP can provide useful information on the development of cardiac failure and pulmonary oedema during weaning. While available data are insufficient to recommend routine insertion of PAC for the only purpose of monitoring PAOP during weaning, its use should be considered when a cardiac origin of failure is highly suspected and/or echographic windows are poor.

### 3.2. Ultrasonography

#### 3.2.1. Cardiac Ultrasonography

Cardiac ultrasound (US) parameters have been widely evaluated as possible predictors of weaning success. In their prospective observational study, Moschietto and colleagues observed that, during the SBT, the maximum speed reached by mitral E’ wave was greater in patients who were successfully weaned from the ventilator [23]. According to the authors, the reduced E’ velocity in weaning failure patients reflects the inability of the heart to reduce wall stress during the adrenergic stimulus of the SBT, and this may be one of the factors involved in the failure of the respiratory weaning process.

These data seem to be confirmed by a recent meta-analysis by Sanfilippo et al. [18]. The authors analyzed the association between echographic parameters and failed weaning from mechanical ventilation. Notably, only one of the numerous studies included was performed in a cardiology intensive care unit (ICU) [21]. The authors found a significant correlation between indicators of diastolic dysfunction and the inability to wean from the ventilator. In particular, a higher E/E’ ratio, a lower E’ wave, and a higher E wave were all associated with an increased risk of weaning failure. Other studies confirmed the association between indicators of poor diastolic function, namely, E/E’, E/A, mitral flow deceleration time, and the risk of prolonged ventilation [10].

While diastolic dysfunction seems to play a pivotal role in respiratory insufficiency after cardiac surgery, the role of systolic impairment—usually represented as reduced LVEF—remains controversial. In fact, as stated above (cf. Section 1), only some, older studies found a correlation between reduced LVEF and weaning failure, while more recent research could not confirm these findings [4,12,14,16,17,18].

#### 3.2.2. Lung and Diaphragm Ultrasound

Extra-cardiac factors can also make weaning difficult, such as pneumonia, pneumothorax, pleural effusion, or phrenic nerve palsy/injury [2,32,33]. Hence, Mayo et al. claim an extensive use of ultrasonography to assess not only cardiac function but also lung parenchyma, pleura, and diaphragm function [32].

The use of lung ultrasound (LU) to evaluate the condition of the lung at the bedside is now widely used and has had a strong increase after the SARS-CoV-2 pandemic [34].

What can be evaluated with LU is not only the quantity of B lines present per pulmonary field; lung ultrasound score (LUS), which has been proven to be a predictive index of failure of ventilatory weaning; and of the onset of WiPO, but also the state of the lung parenchyma. The presence of atelectasis, or large consolidated areas, and an air bronchogram are all conditions that must be considered when deciding to start SBT [35,36].

The use of ultrasound for assessing the breathing effort to generate sufficient force during SBT is widely used.

Through the US, it is possible to evaluate the diaphragm’s excursion and thickness. Diaphragm ultrasound (DU) has been shown to be useful and accurate in diagnosing diaphragmatic dysfunction with a cutoff of 10–14 mm for diaphragmatic excursion (DE) and 30–36% for diaphragmatic thickening fraction (DTF) [37].

However, the diaphragm is not the only inspiratory muscle involved in ventilation. Especially when it is damaged or weakened, the diaphragm has to work in synergy with other muscles to trigger inspiration. In cases of respiratory distress, the sternocleidomastoid muscles, intercostal, and trapezius are also recruited. One of the major limitations of muscle US is its reproducibility [38].

In a recent review and meta-analysis, Lamas-Alvarez et al. evaluated the information obtained from the LUS, DTF, and DE as a predictive ability on the success of respiratory weaning [39]. They conclude that DTF alone is a good predictor of success, while the poor reproducibility of DE makes it less effective. According to the authors, LUS also seems to be a promising indicator, even though the evidence seems less robust [39].

**Figure 2:**
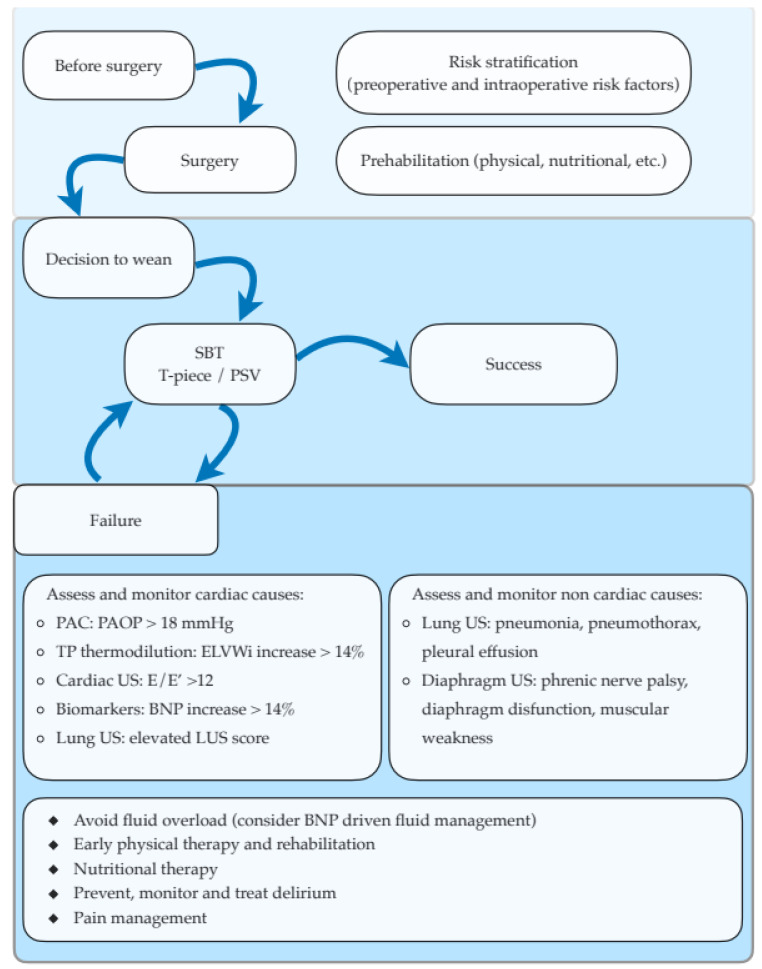
Management algorithm of respiratory weaning after cardiac surgery. After the decision to wean is taken, a SBT is performed by either a T-piece trial or a PSV trial with low or no PEEP. If a SBT fails, possible cardiac and non-cardiac causes of failure need to be assessed and actively monitored during subsequent SBTs. Interventions to facilitate weaning include adequate fluid management, early initiation of physical therapy and nutritional support, and prevention and management of both delirium and postoperative pain. BNP, brain-derived natriuretic peptide; PAC, pulmonary artery catheter; PAOP, pulmonary artery occlusion pressure; PEEP, positive end expiratory pressure; PSV, pressure support ventilation; SBT, spontaneous breathing trial; TP trans-pulmonary; US, ultrasound.

A multimodal integrated approach allows the clinician to comprehend the pathophysiological processes of weaning failure (Figure 2) [40].

### 3.3. Cardiac Biomarkers

Cardiac biomarkers may be a useful tool in the evaluation of patients before and during the weaning process. Two recent pooled analyses of studies on BNP and weaning confirmed that higher levels before SBT are predictive of weaning failure [41,42]. In their observational study, Farghaly and coworkers measured BNP before and after 2 h of SBT. However, they found no difference in basal values of BNP between patients able to complete weaning and patients who failed to wean. At the same time, they demonstrated that an increase in BNP greater than 14% during the SBT was significantly correlated with the inability to complete weaning [43]. Moreover, a multicenter randomized trial was performed to assess the use of BNP to guide fluid management during weaning. This study found that a BNP-driven fluid management strategy was associated with greater use of diuretics, more negative fluid balances, shorter time to weaning completion, and an increased number of ventilator-free days [24].

### 3.4. Transpulmonary thermodilution and Extravascular Lung Water

The role of extravascular lung water (EVLW) assessment during weaning was studied by Dres et al. [25]. The authors directly measured EVLW with transpulmonary thermodilution and found that an increase in EVLW greater than 14% during an SBT could diagnose the development of WiPO with 100% specificity [25]. EVLW indexed for predicted body weight was also found to effectively predict the development of clinically relevant postoperative pulmonary oedema following cardiac and major non-cardiac surgery [44]. In this study, a peak value of indexed EVLW > 14.25 mL/Kg predicted the development of relevant pulmonary oedema with a 72% sensitivity and 81% specificity. Moreover, higher peak values of EVLW were correlated with increased ventilation time and ICU stay, possibly suggesting more difficult weaning in these patients [44]. Estimation of EVLW by the means of lung ultrasound has also been described in cardiac surgery and is discussed in Section 3.2 [45].

EVLW could represent a useful indicator, especially during SBT. However, only a few studies were published on its use in the weaning process, especially after cardiac surgery. Moreover, a PAC is often preferred to transpulmonary thermodilution devices in complex cardiac surgery patients, and thus EVLW is often not available [46]. Hence, we agree with Monnet et al. that a transpulmonary thermodilution device should not be inserted for the only purpose of monitoring weaning. Still, if the device is in place, attention should be paid to EVLW during SBTs [46].

## 4. Ventilatory Strategy

There are many respiratory weaning strategies, and they all pass through spontaneous breathing trials (SBT), but the most effective technique is not yet well defined. The methods most commonly used as daily trials of spontaneous breathing (SB) are T-piece ventilation and pressure support ventilation (PSV) for a time ranging from 30 min to 2 h.

In a randomized clinical trial, Subirà et al. demonstrated that a 30 min SBT of PSV compared to a 2 h SBT with a T-piece resulted in most successful weaning from mechanical ventilation [47].

Recent guidelines to improve early liberation from mechanical ventilation recommended using SBT, early mobilization and physiotherapy, reducing sedation, and the development of protocols [48,49].

An early extubation to non-invasive ventilation did not shorten the time to liberation from any ventilation [50].

Great interest is put into various automatic weaning modes. Automated closed-loop systems can improve the adaptation of mechanical support to patients’ ventilatory needs. These systems continuously monitor changes in ventilation, interpret physiological changes in real time, and adapt ventilation. Closed-loop systems consist of an input that activates the system and an output and a protocol that integrates the two [51]. Despite the extensive development of new systems, a Chocrane review compared automated with traditional trials, the authors conclude by underlining the need for large randomized controlled trials that can effectively compare the efficacy of the two protocols [52].

## 5. Nutrition

Preoperative malnutrition—often defined as low BMI, albumin, or prealbumin levels—is common among cardiac surgery patients and is associated with increased postoperative complications, including prolonged mechanical ventilation [53,54,55,56].

The impact of different nutritional regimens on respiratory weaning has been long evaluated in various studies, starting from the 1980s [57]. In recent years, Huang et al. retrospectively found that patients with a protein intake greater than 1.2 g/kg/day had significantly increased chances of successful weaning [58]. Another recent retrospective trial confirmed that higher calorie and protein intake were associated with better probabilities of successful weaning [59]. In a randomized study, Faramawy and colleagues demonstrated that treating patients with iso-caloric, high-fat, low-carbohydrate enteral feeding could ameliorate ventilation (i.e., reduced carbon dioxide tension) and reduce time on mechanical ventilation [60]. Moreover, early parenteral nutrition was associated with reduced time on mechanical ventilation in patients with contraindications to enteral feeding [61]. Finally, glutamine supplementation, either via the enteral or parenteral route, was associated with reduced time on a ventilator [62].

Unfortunately, none of these studies was conducted in the cardiac surgery setting, where prospective studies on the effect of different nutrition protocols on mechanical ventilation are lacking. Moreover, the aforementioned results may not be translated to the cardiac surgery population, as the mechanisms underlying the need for prolonged ventilation and the metabolic response often differ between cardiac surgery and acute-medical patients [56,63]. Despite these limitations, we believe screening for nutritional risk and prompt initiation of adequate nutritional support should be strongly considered in patients with difficult weaning after cardiac surgery.

## 6. Prolonged Ventilation and Tracheostomy

The need for prolonged ventilatory support after cardiac surgery poses the patient at risk for complications related to both prolonged ventilation and oro-tracheal intubation, such as dysphagia, aspiration pneumonia, and neurological complications related to prolonged sedation [64].

Tracheostomy is often performed in such patients, as it is claimed to reduce complications, facilitate nursing, and accelerate weaning by reducing dead space and work of breathing. However, robust evidence of these benefits is scarce. The best timing and technique for tracheostomy after cardiac surgery are still debated as well [65,66,67,68,69].

### 6.1. Selection of Patients and Timing of Tracheostomy

Tracheostomy after cardiac surgery, even if infrequently performed, is associated with poor prognosis, with a 1-year mortality of 60% and survival of less than 16% at 5 years [70]. Identification of patients that could benefit most from this intervention represents a major challenge in clinical practice. Some risk factors for tracheostomy after cardiac surgery have been identified, including heart failure, diastolic dysfunction, respiratory diseases, renal replacement therapy, emergency surgery, need for mechanical circulatory support, and re-doing surgery [19,70]. Moreover, independent predictors of mortality after tracheostomy in cardiac surgery patients were the duration of ventilation before tracheostomy and the presence of postoperative heart failure associated with respiratory failure, suggesting that careful evaluation of risks and benefits in these patients is of utmost importance [71]. However, evidence on this topic is insufficient, and further, prospective studies are warranted to identify patients that are most likely to benefit from tracheostomy.

The optimal timing for tracheostomy after cardiac surgery is also a matter of debate. Okada et al., in a retrospective, observational study, found that early tracheostomy (i.e., within seven days from ICU admission) was associated with better clinical outcomes, with lower mortality and morbidity rates [68]. In another retrospective observational study, Affronti et al. found that early tracheostomy (i.e., within 14 days after surgery) in patients undergoing cardiac surgery was associated with a shorter ventilation time and ICU and hospital length of stay but did not result in a lower in-hospital and long-term mortality rate [69]. Other studies confirmed the benefit of early tracheostomy on ventilator-free days and length of stay, but the effect on mortality was inconsistent [72,73].

### 6.2. Tracheostomy Technique

Tracheostomy techniques in these patients include percutaneous, surgical, or mixed techniques [74,75]. Surgical tracheostomy has been widely used and was described to be easily performed also in difficult scenarios such as COVID-19 ICUs during the coronavirus pandemic [76]. Percutaneous techniques have been growing in popularity in the last 20 years, but their ability to improve relevant outcomes is still debated [77,78]. Finally, Molardi et al. proposed a hybrid technique to reduce hemorrhagic and infectious complications, such as sternotomy wound infection, in cardiac surgery patients [75]. Current evidence is insufficient to support one or another technique, as infection rate, mortality, and morbidity do not seem to vary significantly between techniques [66,69].

## 7. Conclusions

Difficult respiratory weaning after cardiac surgery can be due to both cardiac and noncardiac causes, affects a significant number of patients, and is related to poor outcomes. Patients at greater risk for difficult weaning may be identified by preoperative as well as surgical factors. In this high-risk population, the use of hemodynamic and echographic indicators to guide the weaning process may be helpful. The assessment of diastolic function and BNP-guided protocols are the most promising interventions and may reduce the weaning time and improve outcomes. A small number of patients requiring prolonged ventilation may be offered tracheostomy, but evidence to support this intervention is scarce. Evidence suggests tracheostomy performed within 14 days of ICU admission might lead to a better outcome than a “late” tracheostomy. No technique—surgical, percutaneous, or hybrid—was proven superior. More research is warranted in this field in order to provide both clinicians and researchers with prospectively validated scores for risk stratification of patients, which may prelude to randomized controlled trials on ventilatory and non-ventilatory interventions.

## Figures and Tables

**Table 1:** Most recent studies on perioperative risk factors for prolonged mechanical ventilation following cardiac surgery.

Author, Year, Reference	Patients	Preoperative Risk Factors	Intra/Postoperative Risk Factors	Protective Factors
Totonchi et al., 2014 [12]	743 consecutive CPB patients	female sex, hypertension, COPD, CKD	endocarditis surgery, bleeding, inotrope dependency	isolated CABG, duration of surgery <4 h, CPB < 60 min
Murthy et al., 2007 [13]	12,777 consecutive cardiovascular patients (excluding HTX and VAD)	BMI, NYHA class, COPD	aortic surgery, CPB time, bleeding, inotrope dependency, low cardiac index, early postoperative complications *	
Nozawa et al., 2005, [14]	52 patients on prolonged ventilation (mean 10 days) after CPB surgery. †		cardiac complications ‡, RRT, inotrope dependency	
Cislaghi et al., 2007, [15]	3269 on-pump CABG patients	LVEF < 30%	re-do surgery, CPB > 90 min, PRBC transfusion, FFP transfusion	
Reddy et al., 2007, [16]	12,662 consecutive cardiac surgery patients (both on- and off-pump)	age, FEV1 < 70%, current smoker, serum creatinine, peripheral vascular disease, LVEF < 30%, recent MI, preoperative ventilation	re-do surgery, urgent-emergent surgery, mitral valve surgery, aortic surgery, use of CPB	
Wang et al., 2022, [19]	5323 CPB patients	age, renal failure, DM, COPD, preoperative pulmonary oedema	combined CABG + valvular surgery, aortic surgery, emergent surgery	

* Stroke, bacteremia, sepsis, or reoperation for bleeding occurring < 72h from surgery. † Study evaluated risk factors for weaning failure from prolonged ventilation. ‡ Low cardiac output syndrome, bleeding, arrhythmias, or cardiac arrest. BMI, body mass index; CABG, coronary artery bypass graft; CKD, chronic kidney disease; COPD, chronic obstructive pulmonary disease; CPB, cardiopulmonary bypass; DM, diabetes mellitus; FEV1, first second forced expired volume; FFP, fresh frozen plasma; LVEF, left ventricular ejection fraction; MI, myocardial infarction; NYHA, New York Heart Association; PRBC, packed red blood cells; RRT, renal replacement therapy; VAD, ventricular assist device.

## Data Availability

Not applicable.
